# Synthesis of NIR-II Absorbing Gelatin Stabilized Gold Nanorods and Its Photothermal Therapy Application against Fibroblast Histiocytoma Cells

**DOI:** 10.3390/ph14111137

**Published:** 2021-11-09

**Authors:** Adewale Oladipo, Thabang Calvin Lebepe, Sundararajan Parani, Rodney Maluleke, Vuyelwa Ncapayi, Grace It Mwad Mbaz, Sandile Phinda Songca, Tetsuya Kodama, Oluwatobi Samuel Oluwafemi

**Affiliations:** 1 Department of Chemical Sciences, Doornfontein Campus, University of Johannesburg, Johannesburg 2028, South Africa; 201504949@student.uj.ac.za (A.O.); calvyn.tl@gmail.com (T.C.L.); sbarani416@gmail.com (S.P.); rodney.maluleke@gmail.com (R.M.); vuyelwa.tito.ncapayi530@gmail.com (V.N.); gmbazitmwad@gmail.com (G.I.M.M.); 2 Centre for Nanomaterials Science Research, University of Johannesburg, Johannesburg 2028, South Africa; 3 Department of Chemistry, University of KwaZulu-Natal, Private Bag X 54001, Durban 4000, South Africa; songcaS@ukzn.ac.za; 4 Graduate School of Biomedical Engineering, Tohoku University, 4-1 Seiryo, Aoba, Sendai 980-8575, Japan

**Keywords:** gold nanorods, gelatin, near-infrared, stability, cytotoxicity, photothermal therapy

## Abstract

The excellent photothermal properties of gold nanorods (Au-NRs) make them one of the most researched plasmonic photothermal nanomaterials. However, their biological applications have been hampered greatly due to surfactant-induced cytotoxicity. We herein report a simple synthesis of highly biocompatible gelatin stabilized Au-NRs (gelatin@Au-NRs) to address this issue. The optical and structural properties of the as-synthesized gelatin@Au-NRs were investigated by Zetasizer, Ultraviolet-Visible-Near Infrared (UV-Vis-NIR) spectroscopy, high-resolution transmission electron microscopy (HR-TEM), and Fourier transform infrared spectroscopy (FTIR). The as-synthesized gelatin@Au-NRs were highly crystalline and rod-like in shape with an average length and diameter of 66.2 ± 2.3 nm and 10 ± 1.6 nm, respectively. The as-synthesized gelatin@Au-NRs showed high stability in common biological media (phosphate buffer saline and Dulbecco’s Modified Eagle’s Medium) compared to CTAB capped Au-NRs. Similarly, the gelatin@Au-NRs showed an improved heat production and outstanding cell viability against two different cancer cell lines; KM-Luc/GFP (mouse fibroblast histiocytoma cell line) and FM3A-Luc (breast carcinoma cell line) compared to CTAB capped Au-NRs and PEG@Au-NRs. An in vitro photothermal therapy study against KM-Luc/GFP showed that gelatin@Au-NRs effectively destroys the cancer cells.

## 1. Introduction

Plasmonic photothermal therapy (PPTT) is a minimally invasive treatment that depends on light irradiation, and it has been widely endorsed for therapeutic application [1]. Noble metallic nanomaterials such as gold nanorods (Au-NRs) are the most studied materials in PPTT due to their surface plasmon resonance (SPR) modes which can be tuned between the first and second near-infrared window [2,3,4,5]. Au-NRs absorbing in the second near-infrared (NIR-II) window (1000–1100 nm) are highly desirable to treat deep-seated tumors [3,5]. However, surfactant-induced cytotoxicity of Au-NRs is a major limitation. The most commonly used surfactant in AuNRs synthesis is hexadecyltrimethylammonium bromide (CTAB). CTAB is used as a soft template in the seed-mediated method. However, studies have shown that it is one major cause of AuNRs toxicity [6,7,8,9]. Therefore, it is essential to find biocompatible, non-toxic, and stable surface modifying agents to passivate Au-NRs and reduce their toxicity whilst maintaining their optical properties [10,11,12]. Many types of surface modifications of Au-NRs by different species such as synthetic polymers [3,13,14,15,16], MXenes [17], Oxides [7,18,19], biopolymers [20,21], proteins [22,23,24], and organic molecules [25] have been reported for biomedical applications. Though some of these surface modifications are very effective, the procedures are complex, expensive, unstable, and often time-consuming [19]. Polyethylene glycol (PEG) polymers are the commonly used Au-NRs surface modifier; however, it has been shown to affect cellular uptake and trafficking of the Au nanoparticles.

On the other hand, gelatin is a versatile natural biopolymer commonly used in biological applications; owing to its biocompatibility, biodegradability, and affordability with several active functional groups, it gains an interest in metal nanoparticles surface modification [26,27,28,29,30]. The use of gelatin for coating gold nanostructures have been reported to significantly reduce (i) plasmon shift of localized surface plasmon resonance (LSPR) and (ii) decrease in absorbance associated with heating during irradiation due to its high thermal conductivity [27,31]. The gelatin layer on the nanorods could also suppress the surface diffusion of atoms during the heating process [11]. However, to the best of our knowledge, there has been no report available in the literature on cytotoxicity, biological stability, and photothermal profiling of gelatin-coated NIR-II absorbing Au-NRs. Herein, we report the synthesis of CTAB capped Au-NRs absorbing in the NIR-II window followed by gelatin passivation to obtain an efficient and highly stable gelatin@Au-NRs in biological media. The photothermal efficiency, cytotoxicity, and phototoxicity of the optimized gelatin@Au-NRs was tested against Fibroblast histiocytoma KM-Luc/GFP cell lines which are stable cells expressing a fusion of luciferase (Luc) and green fluorescent protein genes (GFP) [4]. The as-synthesized material was shown to be biostable and biocompatible, with excellent photothermal properties as a potential tool for application in deep tumor therapy.

## 2. Results and Discussion

### 2.1. Synthesis and Characterization of Gelatin@Au-NRs

**Scheme 1:**
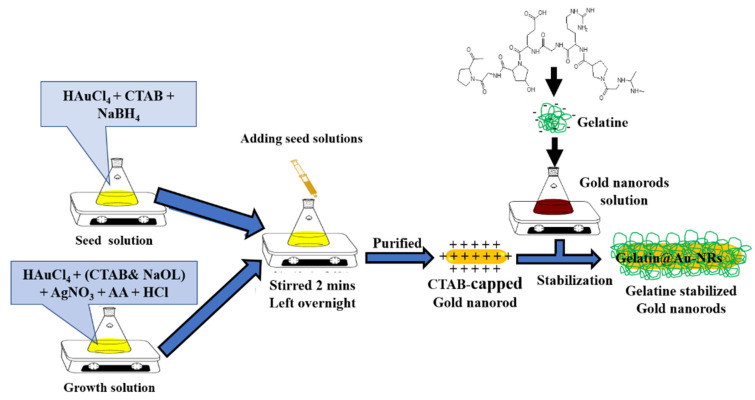
Schematic diagram illustrating the gelatin stabilization of gold nanorods.

The seed-mediated method was used to synthesize CTAB-capped Au-NRs and gelatin@Au-NRs (Scheme 1). The as-synthesized CTAB-capped Au-NRs were characterized using Zetasizer, TEM, and UV-Vis-NIR. The absorption spectrum shows that they are absorbed in the NIR-II window with absorption at 1045 nm (Figure S1). The dynamic light scattering (DLS) results show that they are anisotropic particles with two peaks at 8.72 ± 2.98 nm and 78.82 ± 10.66 nm (Figure S2). TEM confirmed that they are indeed rod-like in shape with an average length and diameter of 65.2 ± 2.4 nm and 10.1 ± 0.35 nm, respectively. Gelatin coating on Au-NRs was achieved by mixing gelatin at different volumetric ratios to Au-NRs (10:1, 4:1, 2:1, and 1:2), using a magnetic stirrer (400 rpm @ room temperature, Hei-Tec with Pt1000 temperature sensor (V4A) Magnetic Stirrers, Heidolph Instruments GmbH & Co. KG, Schwabach, Germany). The UV-Vis-NIR spectra (Figure S3) showed that coating with gelatin at the ratios of 10:1 and 4:1 caused a 7 nm and 4.5 nm blue-shift in the parent Au-NRs LSPR wavelength peak, respectively, while at a 2:1 ratio, a slight shift of 0.5 nm was observed. However, at a 1:2 ratio, there was a redshift in the LSPR peak position from 1045 nm to 1052.5 nm. The DLS results show that the blue shift observed in the absorption spectra at ratios 2:1, 4:1, and 10:1 was due to the reduction in the length of the AuNRs during coating (Figure S2).

### 2.2. Stability of Gelatin@Au-NRs in Biological Media

**Figure 1:**
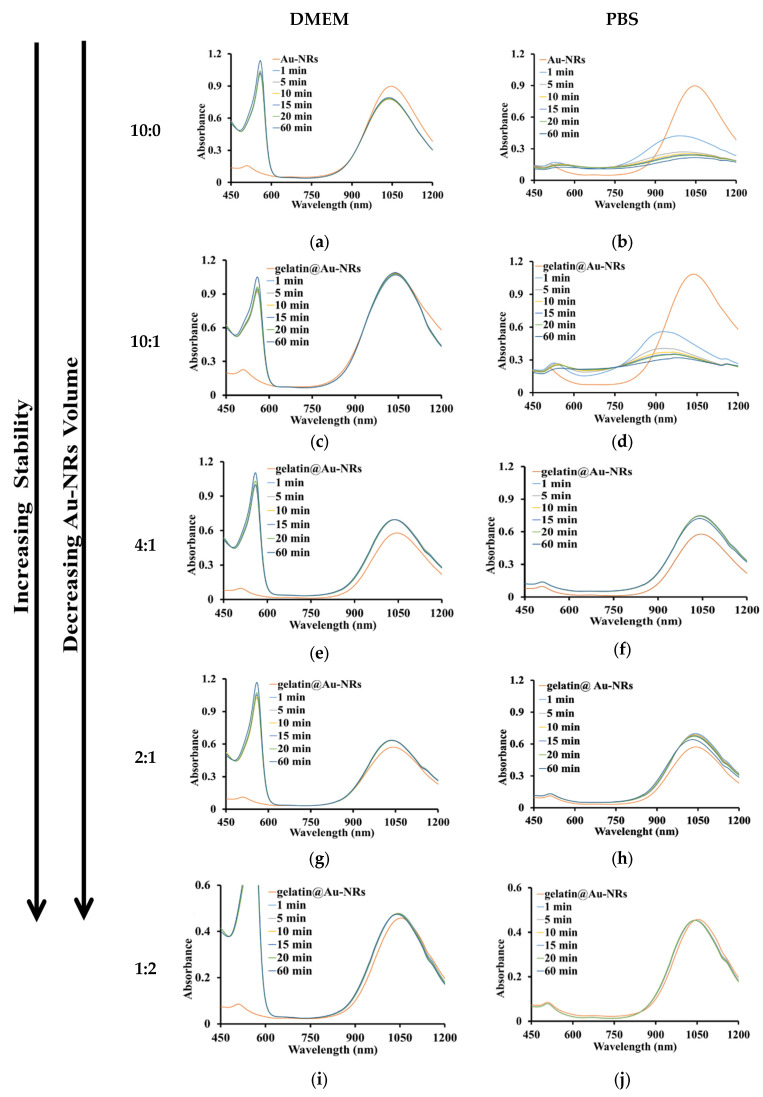
Absorption spectra of CTAB-capped AuNRs (**a**,**b**) and gelatin@Au-NRs at different volumetric ratios (**c**–**j**) in DMEM medium and PBS (pH 7.4) solution.

One of the most important parameters in using nanomaterials for biological application is their stability in biological medium. For this study, two common media were used, namely, DMEM culture medium and PBS at pH 7.4. Gelatin@Au-NRs at different volumetric ratios were added to both media at 37 °C, and their stability over time was monitored using a UV-Vis-NIR spectrophotometer. The changes in LSPR absorbance and wavelength were used to explain the stability [11]. When CTAB-capped Au-NRs were dispersed in a culture DMEM medium, it immediately showed a blue shifting in its LSPR position with a reduction in absorbance within the first minute (Figure 1a). Then, it became stable onwards with no significant changes.

However, the gelatin@Au-NRs (at volume ratio 10:1) dispersed in the DMEM medium exhibited no significant changes in LSPR position and absorbance with time (Figure 1c). The absorption peak seen at 561 nm is attributed to the phenol red indicator in the DMEM (Figure S5). The stability of CTAB-capped Au-NRs and gelatin@Au-NRs (10:1) were also examined in the PBS (pH 7.4) solution, and both resulted in a significant blue shifting from NIR-II to NIR-I and a decrease in the LSPR peak absorbance (Figure 1b,d). After 5 min, the LSPR peak wavelength was shifting slightly with a reduction in absorbance as the incubation time increased. This could be attributed to the ionic effect induced by the various ion components in the PBS (pH 7.4) solution. 

It is believed that effective biopolymer protection of nanoparticles should prevent the aggregation process via electrostatic or steric repulsion [10,11,12]. However, the 10:1 ratio result showed a different outcome as they are less stable in PBS (pH 7.4). Therefore, we investigated the optimum concentration that would be required to stabilize the Au-NRs by using gelatin as the capping agent. To do this, we optimized the volumetric ratios of Au-NRs and gelatin dispersed in a biological medium. The UV-Vis-NIR spectra of the gelatin@Au-NRs obtained at ratios 4:1 and 2:1 in the DMEM medium (Figure 1e,g) showed a slight blue shift in the LSPR wavelength position with enhanced absorbance at 1 min, and after that, the absorbance remained unchanged. Similarly, an increase in absorbance was observed in the PBS (pH 7.4) medium for both ratios but with a slight shift (Figure 1f–h). In contrast to the results obtained at a 10:1 ratio, where LSPR absorbance decreased as early as 5 min in PBS (pH 7.4), increasing the gelatin volume provides better and improved stability at ratios 2:1 and 4:1. At a 1:2 ratio, the gelatin@Au-NRs demonstrated stability in the DMEM medium with slightly enhanced plasmon absorbance (Figure 1i) over time. In PBS (pH 7.4) medium, the gelatin@Au-NRs at a 1:2 ratio showed good stability. The LSPR absorbance remained unchanged with a slight reduction of about 3 nm in the wavelength position (Figure 1j). The 1:2 ratio result demonstrated a significant improvement when compared with other ratios. Such high stability is critical for reducing nonspecific binding in biological systems.

**Figure 2:**
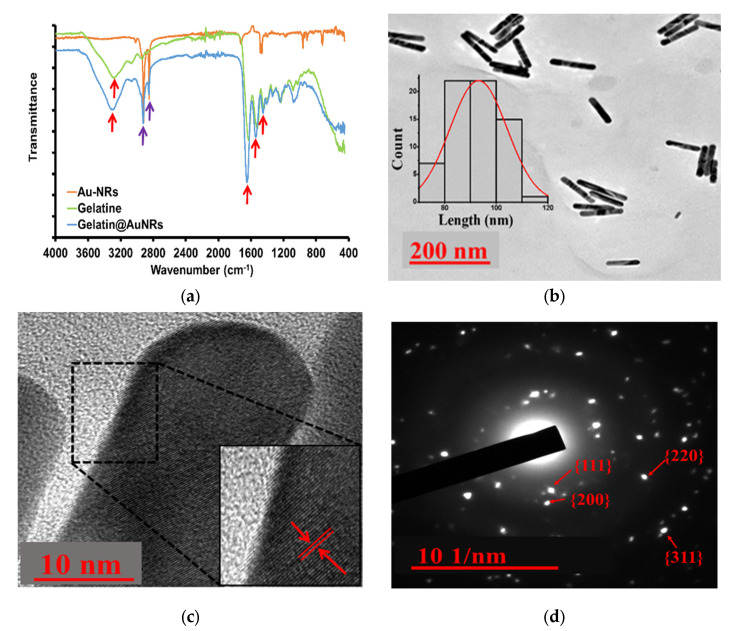
(**a**) FTIR, (**b**)TEM image with corresponding length distribution histogram, (**c**) HRTEM and (**d**) SAED of gelatin@Au-NRs.

Since gelatin@Au-NRs (1:2) gave the best stability result, it was further characterized using FTIR, HRTEM, and zeta potential. Figure 2a shows the FTIR spectra of CTAB-capped AuNRs, gelatin, and gelatin@Au-NRs. The FTIR spectrum of gelatin@Au-NRs shows gelatin N-H stretching coupled with hydrogen bonding and free O-H between 3326–3026 cm^−1^. The strong C = O and medium C-N stretching vibrations of gelatin can also be seen at 1655 and 1549 cm^−1^, respectively. All these peaks appeared at a slightly higher wavenumber from that of bare gelatin, and this could be attributed to the interaction of CTAB C-N^+^ on the surface of Au-NRs with the gelatin. In addition, the CTAB -CH_2_ stretching peaks were maintained in the gelatin@Au-NRs. The TEM micrograph (Figure 2b) shows that the Au-NRs after gelatin-coating (gelatin@Au-NRs) are rod-like in shape with an average length and diameter of 66.2 ± 2.3 nm and 10 ± 1.6 nm, respectively. The aspect ratio was calculated to be 6.62. This shows that there was no noticeable morphological change in comparison with bare Au-NRs. The HRTEM image (Figure 2c) provides further insight into the microstructure (shape) and crystallinity of the as-prepared gelatin@Au-NRs. The HRTEM confirms that the as-synthesized materials are rod-like in shape, while the presence of lattice fringes confirmed their high crystallinity. The measured lattice spacing (d) of 0.20 nm corresponds to the {111} lattice plane of Au (JCPDS card No. 04–0784). In addition, the presence of bright spots in the selected area electron diffraction (SAED Figure 2d) further confirmed the highly crystalline nature of the gelatin@Au-NRs. The diffraction pattern corresponds to {111}, {200}, {220}, and {311} reflections of face cubic centre Au, which showed that the crystallinity of the Au-NRs was not affected by gelatin coating. Furthermore, the zeta potential changes from + 42.13 mV (CTAB-capped Au-NRs) to + 0.23 mV after gelatin coating. These results confirmed the successful formation of gelatin@Au-NRs. The gelatin@Au-NRs showed good colloidal stability in water for over 1 year (Figure S4).

**Figure 3:**
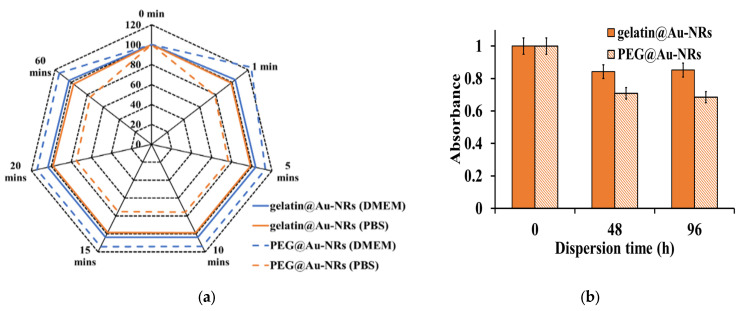
(**a**) Absorption radar graph of gelatin@Au-NRs at volumetric ratio 1:2 and PEG@Au-NRs in PBS (pH 7.4) solution and DMEM culture medium at different incubation times. (**b**) comparison of LSPR absorbance between PEG@Au-NRs and gelatin@Au-NRs (1:2) dispersed in PBS (pH 7.4) solution, at 0 h, 48 h and 96 h.

PEG has been widely used to stabilize AuNRs [32], so we prepared PEGylated Au-NRs and compared their stability with gelatin@Au-NRs produced at a 1:2 ration using a radar plot. Figure 3a represents the absorbance of gelatin@Au-NRs (1:2) and PEG@Au-NRs. The results show that both materials were stable for 60 min in the DMEM medium. The stability of gelatin@Au-NRs (1:2) compared to PEG@Au-NRs in PBS (pH 7.4) showed different results. The absorbance of PEG@Au-NRs was around the 80% radar mark, which is lower when compared to that of gelatin@Au-NRs, which is >90% at 60 min. The absorption of PEG@Au-NRs and gelatin@Au-NRs (1:2) were measured after 48 h and 96 h in PBS (pH 7.4) to observe the effectiveness of the gelatin coating with respect to PEG.

The result shows that after 48 h, gelatin@Au-NRs absorbance was decreased by 14.7%, while that of PEG@Au-NRs was decreased by 29.2% compared to the initial absorbance. After 96 h, both the PEG@Au-NRs and gelatin@Au-NRs showed a negligible decrease in absorbance intensity when compared with the 48 h results (Figure 3b). This shows that in PBS (pH 7.4), the as-synthesized gelatin@Au-NRs (1:2) maintained better LSPR absorbance and position compared to PEG@Au-NRs after 48 h. Thus, it can withstand extreme chemical and biological conditions.

### 2.3. Photothermal Profiling Analyses

Photothermal profiling of gelatin@Au-NRs (1:2) was performed by irradiating it with a NIR laser at (0.5 W/cm^2^, 1064 nm) at different time intervals (0.5 to 3 min) and different concentrations (10 to 100 µg/mL) in a 48-well plate. The temperature changes (ΔT) were monitored using a thermocouple probe inserted at the midpoint of the solution. The heat generated by the gelatin@Au-NRs in an aqueous solution under laser irradiation was observed to be concentration-dependent. 

**Figure 4:**
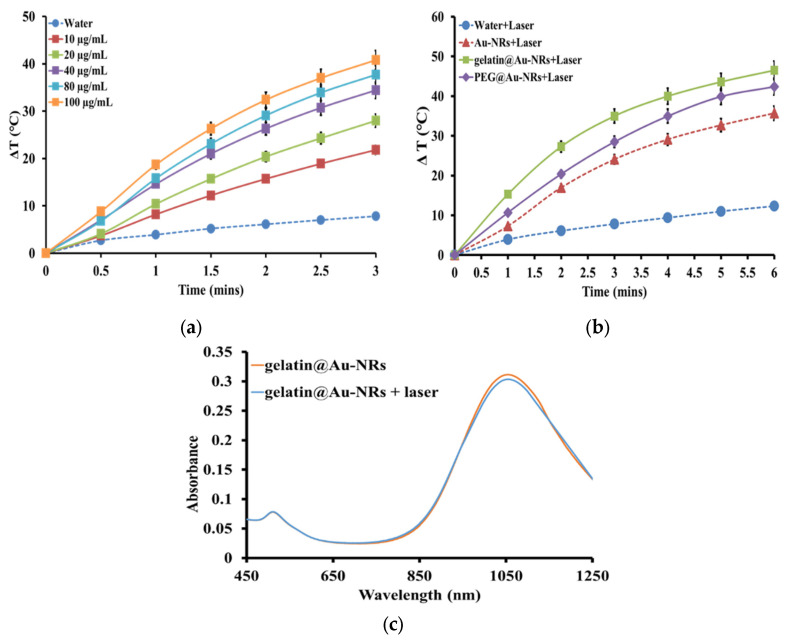
Photothermal response (**a**) temperature change at different concentrations of gelatin@Au-NRs (1:2) after exposure to NIR laser (1064 nm, 0.5 W/cm^2^) for 3 min. (**b**) Change in temperature under higher irradiation power at 1064 nm, 0.9 W/cm^2^ for 6 min at the same concentration of 80 µg/mL. (**c**) Absorption spectra of 80 µg/mL gelatin@Au-NRs (1:2) measured before and after laser irradiation (1064 nm, 0.9 W/cm^2^) for 6 min.

The laser irradiation of water showed a temperature change of less than 10 °C when irradiated for 3 min. In contrast, different concentrations of gelatin@Au-NRs from 10 µg/mL to 100 µg/mL at 3 min, showed ΔT increases from 21 to 42 °C (Figure 4a). For comparison, the ΔT were also studied for CTAB capped Au-NRs and PEG@Au-NRs. As shown in Figure 4b, laser irradiation of water shows a ΔT of about 10 °C when irradiated for 6 min, whereas laser irradiation of CTAB-Au-NRs, gelatin@Au-NRs, and PEG@Au-NRs under similar experimental condition, shows ΔT of 35 °C, 46 °C, and 41 °C, respectively. The results show that the gelatin biopolymer improved the thermal response of the Au-NRs with higher photothermal conversion efficiency than PEG@Au-NRs and CTAB capped Au-NRs. To evaluate the photostability of gelatin@Au-NRs, laser irradiation was performed at the wavelength of 1064 nm, at 0.9 W/cm^2^ for 3 min and analyzed using a UV-Vis-NIR spectrophotometer. The result demonstrated that no significant change was observed in the absorption spectrum of the gelatin@Au-NRs before and after irradiation (Figure 4c). The absorbance and LSPR maximum peak were almost the same even after irradiation at higher laser power, indicating that the gelatin@Au-NRs are photothermally stable enough to be employed as an efficient photothermal agent.

### 2.4. Cell Viability and Photothermal Performance Analyses

**Figure 5:**
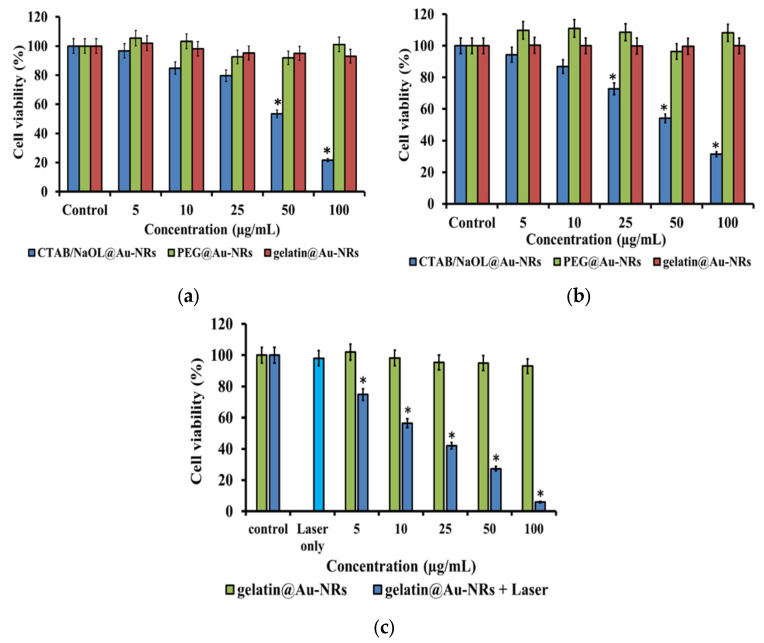
Cellular viability of CTAB@ Au-NRs, gelatin@ Au-NRs, and PEG@ Au-NRs on (**a**) KM-Luc/GFP cells (**b**) FM3A-Luc; at different concentrations. (**c**) In vitro laser cytotoxicity of gelatin@Au-NRs (1:2) at different concentrations under irradiation (1064 nm, 0.5 W/cm^2^) for 3 min on KM-Luc/GFP cell lines (* = *p* < 0.05).

The biocompatibility of gelatin@Au-NRs was evaluated to ascertain its potential as a photothermal agent for cancer therapy. KM-Luc/GFP cells (mouse fibroblast histiocytoma cell line) and FM3A-Luc cells (breast carcinoma cell line) were used for this study. A standard MTT cellular viability assay was used to evaluate the cell viability. The cell viability of gelatin@Au-NRs was also compared with that of CTAB capped Au-NRs and PEG@Au-NRs. Figure 5a,b showed that the cell viability of both gelatin@Au-NRs and PEG@Au-NRs on KM-Luc/GFP and FM3A-Luc were higher than 90% even at higher concentrations of nanorods, while CTAB capped Au-NRs were more toxic at high concentration (>25 µg/mL).

These results validated that gelatin@Au-NRs offers excellent biocompatibility in the given concentration range, which is essential for biomedical applications. It is similar to the pegylated AuNRs, which are widely used [32]; however, gelatin is cheaper than PEG. Moreover, due to the outstanding photothermal profiling results of gelatin@Au-NRs over PEG@Au-NRs, the anti-cancer photothermal property of gelatin@Au-NRs was investigated on KM-Luc/GFP cell lines at different concentrations over 24 h incubation, followed by laser irradiation. The result (Figure 5c) shows that at 5 µg/mL, there was a 25% reduction in cell survival. This increased to a 94% reduction at 100 µg/mL, indicating that the gelatin@Au-NRs possess an excellent ability to destroy cancer cells and inhibit their proliferation. The experiment was also repeated with a laser alone, without the gelatin@Au-NRs. The result shows no reduction confirming that the laser alone was not responsible for the cell lethality. The NIR-II gelatin results were demonstrated to be more effective than other NIR-II absorbing photo agents such as Fe_3_O_4_@CuS-PEG [33] and the scaffold Au-NRs and Au nanostars with only 92% after irradiation [29].

## 3. Materials and Methods

### 3.1. Materials

Hydrogen tetrachloroaurate hydrate (HAuCl_4_.xH_2_O, 99.9%), sodium borohydride (NaBH_4_ 99%), silver nitrate (AgNO_3_, 99%), hexadecyltrimethylammonium bromide (CTAB, ≥99%), ascorbic acid (99%), sodium oleate (NaOL, ≥99%), Methoxypolyethylene glycol thiol (mPEG-SH) (MW 5000 Da), Dulbecco’s Modified Eagle’s Medium (DMEM), phosphate saline buffer (PBS, Ca^2+^ and Mg^2+^ free, pH = 7.4), and 3-(4,5-Dimethylthiazolyl-2)-2,5-diphenyl tetrazolium bromide (MTT) were purchased from Sigma-Aldrich, South Africa and Japan. Hydrochloric acid (HCl, ACS reagent 37 wt.%) was obtained from Wako. Gelatin from porcine skin was obtained from Fluka, Japan. All chemicals were of analytical grade and were used without further purification. All glassware used in the experiment was cleaned and washed thoroughly with MilliQ water (18.2 MΩ cm @ 25 °C) and dried before use.

### 3.2. Synthesis of Gold Nanorods

Au-NRs were synthesized following the previously reported procedure with a slight modification [34]. In brief, 364.50 mg of CTAB powder was added to 10 mL distilled water and heated to 40 °C. A total of 250 µL HAuCl_4_ solution (0.01 M) was added to the solution followed by gentle stirring for 30 min. A freshly prepared 600 µL of ice-cold 0.01 M NaBH_4_ was then added, followed by vigorous stirring for 2 min. The resultant light brown solution, which serves as the Au seed solution, was kept at 30 °C for 30 min before use.

The growth solution was prepared in a separate flask by adding 3500 mg of CTAB to 617 mg NaOL in warm water (40 °C). Then, 12.4 mL of 4 mM AgNO_3_ was added and left undisturbed for 15 min, after which 125 mL of 1 mM HAuCl_4_ was added. The resulting mixture was stirred for 60 min until it became colorless. After that, 3.4 mL of HCl (37 wt.% in water) and 625 µL of 0.064 M ascorbic acid were added, and the solution was vigorously stirred for 1 min. The rod growth process was initiated by adding 125 µL of the seed solution to the growth solution, and the whole solution was stirred for 0.5 min and left undisturbed at 30 °C overnight. The Au-NRs obtained in the solution were then centrifuged at 7000 rpm for 30 min (Sigma 3-30K, Sigma Centrifuges GmbH, Osterode am Harz, Germany) to remove excess unreacted reagents. The pellets which precipitated were carefully pipetted out and redispersed in MilliQ water. No size and/or shape-selective separation was performed.

### 3.3. Preparation of Gelatin@Au-NRs

The preparation of gelatin@Au-NRs was achieved in a one-step process by simply mixing gelatin solution (dissolved in warm water) with the as-synthesized gold nanorods. The different volumetric ratios of nanorods to gelatin solution (10:1, 4:1, 2:1, and 1:2) were prepared and placed on a magnetic stirrer (400 rpm @ room temperature, Hei-Tec with Pt1000 temperature sensor (V4A) magnetic stirrers, Heidolph Instruments GmbH & Co. KG, Schwabach, Germany) for 6 h to produce gelatin@Au-NRs. Subsequently, the resulting solution was centrifuged at 9000 rpm for 15 min (Sigma 3-30K centrifuge) to remove any excess gelatin, and the precipitate was redispersed in ultrapure water.

### 3.4. Preparation of Polyethylene Glycol (mPEG-SH) Capped Au-NRs 

The PEG capped Au-NRs (PEG@Au-NRs) were obtained by adding 0.5 mL of 1 mM mPEG-SH (MW 5000 Da) and 0.1 mL of PBS (phosphate buffer saline, pH = 7.4) to 0.5 mL of previously synthesized Au-NRs under magnetic stirring for 24 h. The mPEG-SH capped Au-NRs were obtained after centrifugation (Sigma 3-30K centrifuge) to remove any access mPEG-SH followed by sonication (Professional—Ultrasonic cleaner EINS SCI, United scientific, Goodwood, South Africa) at 13,000 rpm for 10 min. The PEG@Au-NRs were dispersed in ultrapure water for further studies.

### 3.5. Characterization

The optical absorption spectra of all samples were recorded in a 10 mm rectangular micro quartz cell using a JASCO V-770 Ultraviolet-Visible-Near Infrared (UV-Vis-NIR) spectrophotometer (JASCO, Tokyo, Japan). Fourier transform infrared spectroscopy (FT-IR) spectra were recorded using FT-IR (Spectrum two UATR spectrometer, Perkin Elmer, UK) in the spectral region 4000 to 400 cm^−1^ at 1 cm^−1^ resolution. High-resolution transmission electron microscopy (HRTEM) imaging was performed on a JEOL 2010 operating at 200 kV (JEOL, Japan). The colloidal solutions’ effective surface charge and dynamics before and after modification were measured using a Photal ELS-Z2MH instrument (Otsuka, Tokyo, Japan). NIR irradiation was made with a continuous Nd: YV04 air-cooled laser (1064 nm; TEM_00_ beam diameter, 0.6 mm; CYD-010-TUBC; Neoarc).

### 3.6. Biological Medium Stability Studies

The gelatin@Au-NRs were dispersed in complete cell culture medium DMEM (Dulbecco’s Modified Eagle Medium supplemented with 10% fetal bovine serum containing 1% L-glutamine-penicillin-streptomycin and 0.5% Geneticin G418) (Sigma, Kanagawa, Japan) and PBS (Ca^2+^ and Mg^2+^ free, pH 7.4) (Sigma, Japan) in a cuvette holder. The changes in absorption spectra with time were monitored using a JASCO V-770 UV-Vis-NIR spectrophotometer.

### 3.7. Photothermal Profiling Study 

Gelatin@Au-NRs of different concentrations (5, 10, 25, 50, and 100 µg/mL) in a 1.5 mL Eppendorf tube were irradiated with a 1064 nm NIR laser (0.5 W/cm^2^) for 3 min. Distilled water was used as the control. The temperature of the solutions was measured by a thermocouple (KEYENCE, PC Link High Function Recorder, GR-3500 Series, KEYENCE Co., Ltd., Osaka, Japan). In another reaction, 80 µg/mL of CTAB-capped Au-NRs, gelatin@Au-NRs, and PEG@Au-NRs were prepared in a 1.5 mL Eppendorf tube. All the samples were then irradiated under a higher irradiation power at 1064 nm, 0.9 W/cm^2^. The temperature of the solutions was measured by a thermocouple. Gelatin@Au-NRs absorption was measured before and after laser irradiation. The temperature change was calculated using the following Equation (1):

ΔT = T_f_ − T_i_ (1)

where ΔT is temperature change, T_f_ is the final temperature, while T_i_ is the initial temperature. The temperature is given in degrees Celsius (°C).

### 3.8. Cell Culture and In Vitro Cytotoxicity Assay

C3H/He mouse mammary carcinoma cells (FM3A-Luc), which stably express a fusion of luciferase (Luc), and mouse fibroblast histiocytoma cells (KM-Luc/GFP) which stably expresses both (Luc) and enhanced-green fluorescent protein (GFP) (both cells were obtained from the Cell Resource Centre for Biomedical Research, Institute of Development, Aging and Cancer, Tohoku University), were used for cytotoxicity studies. The FM3A-Luc and KM-Luc/GFP cells were cultured in RPMI 1640 and DMEM media at 37 °C in a 5% CO_2_ atmosphere with 70% humidity. The media were supplemented with 10% fetal bovine serum (FBS), 1% L-glutamine-penicillin-streptomycin, and 0.5% Geneticin G418. Cells were split into 48-well plates using a standard trypsin-based method with a final concentration of 5.0 × 10^4^ cells/mL at about 90% confluence. The cells were plated at 500 µL/well and incubated for 24 h. Then, a gelatin@Au-NRs solution dispersed in a complete culture medium at different concentrations (0, 5, 10, 25, 50, and 100 µg/mL) was added to the wells and incubated for another 1 h. The cell culture medium was changed, washed twice with PBS (pH 7.4) to remove the gelatin@Au-NRs that were not taken up by the cells, and replaced with a fresh medium for further 24 h incubation. Cell viability was investigated via a standard (3-(4,5-dimethylthiazol-2-yl)-2,5-diphenyltetrazolium bromide) tetrazolium MTT assay. A PBS (pH 7.4) solution of MTT (5 mg/mL) was added to the wells (50 µL/well) and incubated at 37 °C for 3 h. The media was carefully removed, and dimethyl sulfoxide (DMSO, 300 µL/well) was added to dissolve the formed purple formazan crystals, and the plate was covered with aluminum foil and incubated for 5 min. The absorbance of each well was then measured at 590 nm by a WAKO LS multi-plate reader. The same procedure was repeated for CTAB-capped AuNRs and PEG@Au-NRs.

### 3.9. In Vitro Photothermal Therapy Assay

KM-Luc/GFP cells were seeded in a 48-wells plate at 500 µL/well with a final concentration of 5 × 10^4^ cells/mL at 80% confluence and incubated at 37 °C for 24 h. A gelatin@Au-NRs solution dispersed in a complete culture medium at different concentrations (0, 5, 10, 25, 50, and 100 µg/mL) was added to the wells at 110 µL/well and incubated for 1 h. The cell culture medium was changed to remove the gelatin@Au-NRs that were not taken up by cells, washed twice with PBS (pH 7.4), and replaced with a fresh medium. The cells were irradiated with 1064 nm laser light (0.5 W/cm^2^) for 3 min and further incubated for 24 h. Cell survival was investigated using a standard MTT assay as mentioned above.

### 3.10. Statistical Analysis

Data are presented as the mean ± standard error of the mean (SEM). Statistical comparisons were made using Tukey’s multiple comparisons test. Statistically, values of *p* < 0.05 were significant.

## 4. Conclusions

In summary, we have demonstrated the synthesis of simple and highly biocompatible, gelatin-coated NIR-II absorbing Au-NRs with enhanced photothermal and biological activities for effective inhibition of cancer cell proliferation. The as-synthesized gelatin@Au-NRs maintained better LSPR absorbance and position than PEG@Au-NRs after 48 h in PBS (pH 7.4), which showed that it could withstand extreme chemicals and biological conditions. The Photothermal profiling results showed that the gelatin@Au-NRs displayed higher photothermal conversion efficiency than PEG@Au-NRs and CTAB capped Au-NRs without any change in absorbance and LSPR peak position after irradiation at higher laser power. Hence, they possessed higher photothermal stability. Furthermore, the as-synthesized gelatin@Au-NRs showed comparable high cell viability (>90%) against KM-Luc/GFP and FM3A-Luc cancer cells in comparison with PEG@Au-NRs at higher concentrations with an excellent anti-cancer photothermal property upon laser irradiation even at low concentrations. This as-synthesized gelatin@Au-NRs offers a new type of photothermal agent with an efficient photothermal effect. We believe it will be of prodigious interest for many plasmonic and biomedical applications, especially in photothermal cancer treatment.

## Data Availability

The data is contained in the article and Supplementary Materials.

## Supplementary Materials

The following are available online at https://www.mdpi.com/article/10.3390/ph14111137/s1, Figure S1: Au-NRs UV-Vis-NIR spectrum insert TEM images of Au-NRs. Figure S2: Dynamic light scattering average size distribution of bare-Au-NRs and gelatin-coated Au-NRs at different ratios. Figure S3: UV-Vis-NIR spectrum of gelatin-coated Au-NRs at different volumes. Figure S4: UV-Vis-NIR spectra of gelatin@Au-NRs after synthesis and a year later. Figure S5: UV-Vis-NIR spectra of DMEM only.
