# Supplementary material for: Synthesis of NIR-II Absorbing Gelatin Stabilized Gold Nanorods and Its Photothermal Therapy Application against Fibroblast Histiocytoma Cells
Source: Pharmaceuticals (Basel). 2021 Nov 9;14(11):1137. doi: 10.3390/ph14111137 (PMC8625232; doi:10.3390/ph14111137)
Supplement: Supplementary file 1 [file pharmaceuticals-14-01137-s001.zip › pharmaceuticals-1441581-supplementary.pdf]

# Supporting Information

# Synthesis of NIR-II Absorbing Gelatin Stabilized Gold Nanorods and Its Photothermal therapy Application against fibroblast histiocytoma cells.

**Adewale O. Oladipo [1,2], Thabang C. Lebepe [1,2], Sundararajan Parani [1,2], Rodney Maluleke [1,2],Vuyelwa Ncapayi[1,2], Grace I.M. Mbaz[1,2], Sandile P. Songca [3], Tetsuya Kodama [4*] and Oluwatobi S. Oluwafemi [1,2**]**

[1] Department of Chemical Sciences, University of Johannesburg, P.O. Box 17011, Doornfontein 2028, Johannesburg, South Africa.

[2] Centre for Nanomaterials Science Research, University of Johannesburg, Johannesburg 2028, South Africa.

[3] Department of Chemistry, University of KwaZulu-Natal, Private Bag X 54001, Durban, 4000, South Africa

[4] Graduate School of biomedical engineering, Tohoku University, 4-1 Seiryo, Aoba Sendai, 980-8575, Japan.

Correspondence: kodama@tohoku.ac.jp (T.K); oluwafemi.oluwatobi@gmail.com (O.S.O)

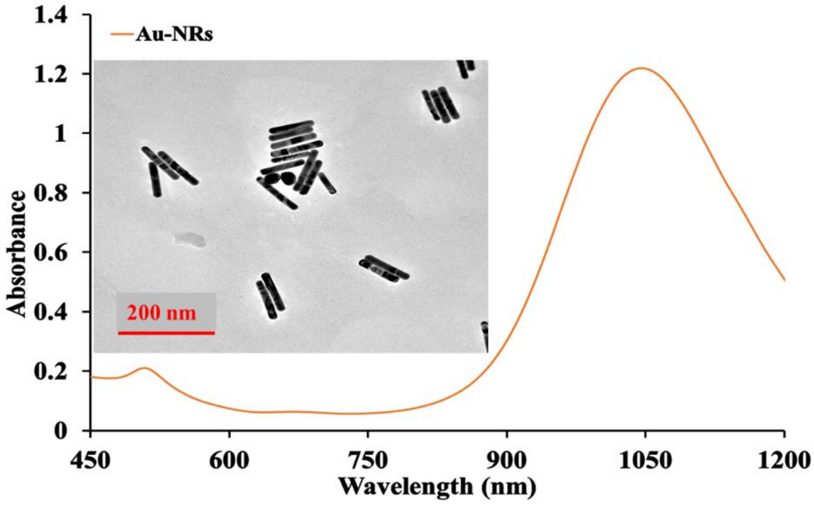


**Figure S1** Au-NRs UV-Vis-NIR spectrum insert TEM images of Au-NRs

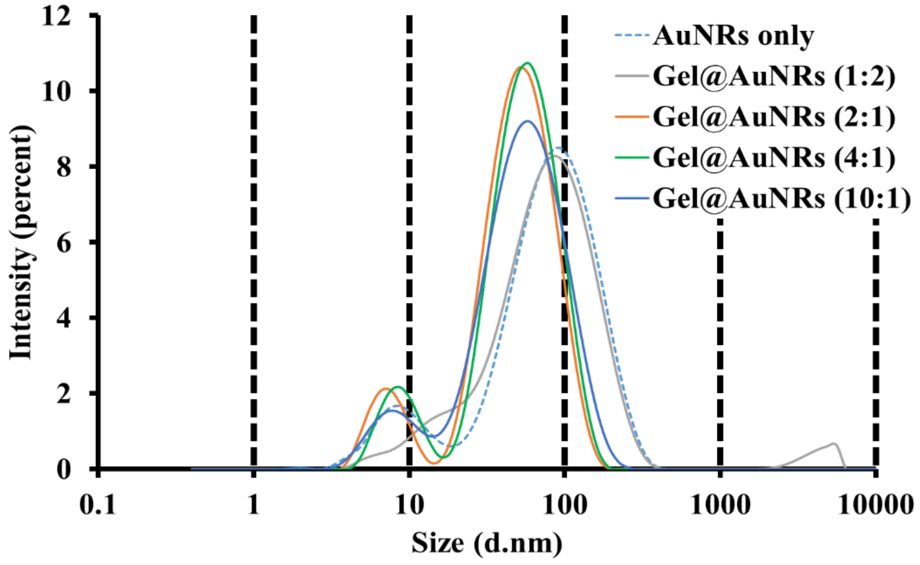


**Figure S2** Dynamic light scattering average size distribution of bare-Au-NRs and gelatin coated Au-NRs at different ratios.

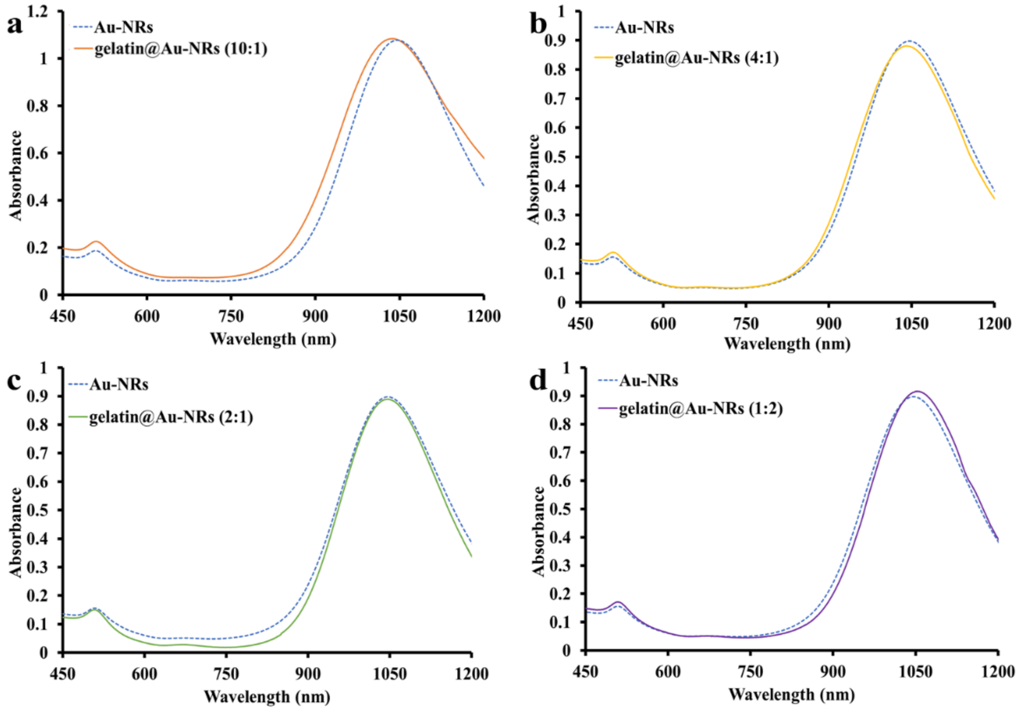


**Figure S3** UV-Vis-NIR spectrum of gelatin coated Au-NRs at different volume.

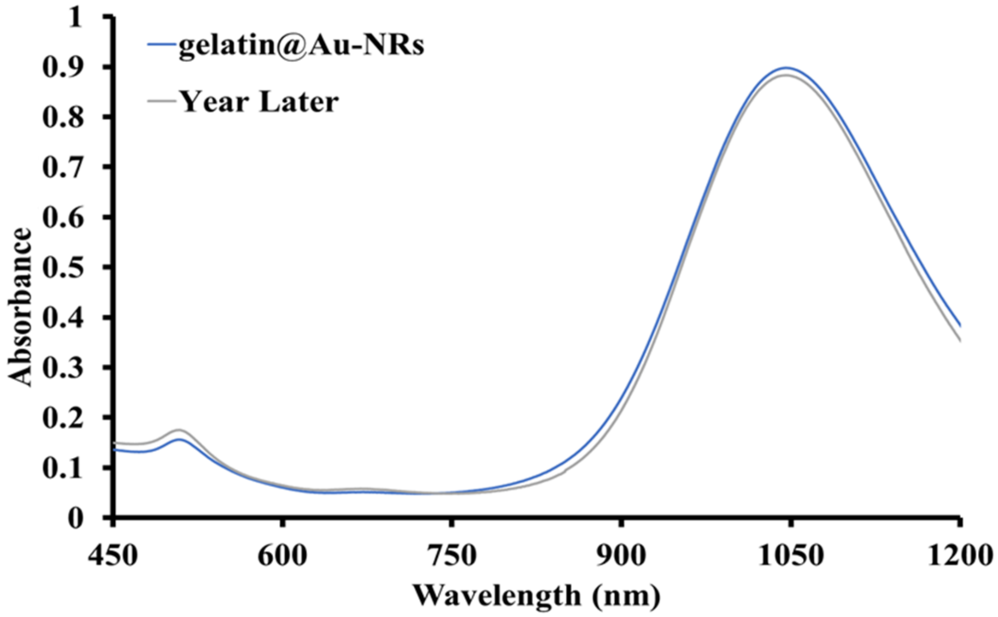


**Figure S4** UV-Vis-NIR spectra of gelatin@Au-NRs after synthesis and a year later

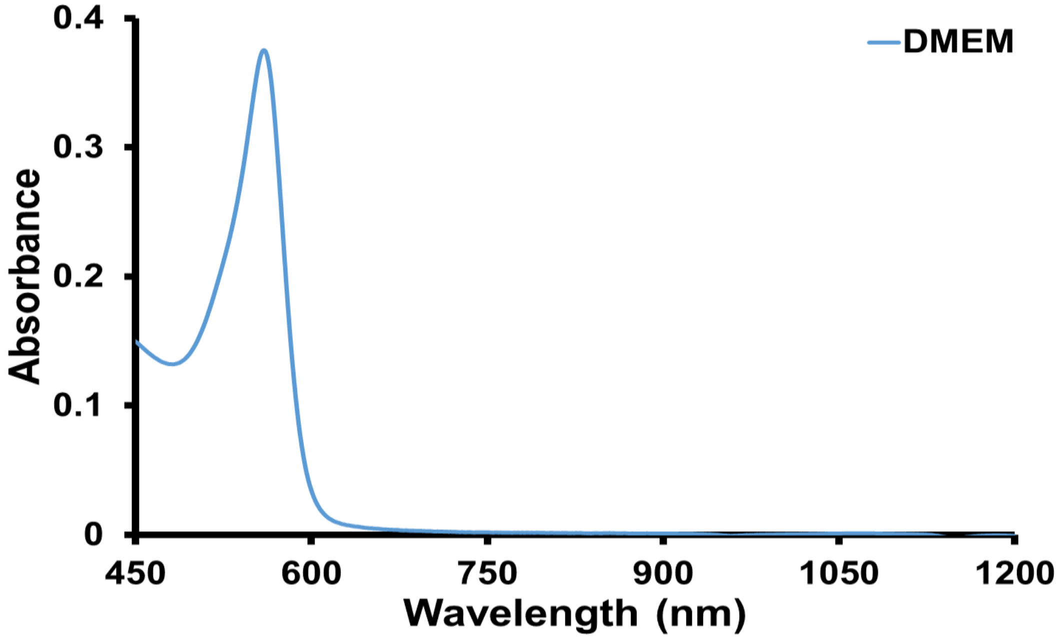


**Figure S5** UV-Vis-NIR spectra of DMEM only
